# Effects of benzylaminopurine and gibberellic acid on growth, yield, and nutrient composition of greenhouse cultivated yellow cherry tomatoes

**DOI:** 10.1038/s41598-025-27667-6

**Published:** 2025-11-12

**Authors:** Rafat A. Eissa, Muziri Mugwanya, Fahad Kimera, Hani Sewilam

**Affiliations:** 1 Center for Applied Research on the Environment and Sustainability (CARES), School of Science and Engineering, The American University in Cairo, AUC Avenue, P.O. Box 74, New Cairo, 11835 Egypt; 2 UNESCO Chair in Hydrological Changes and Water Resources Management, RWTH Aachen University, Aachen, Germany

**Keywords:** Plant growth regulators, Cherry tomatoes, Foliar spray, Hydroponics, Physiology, Plant sciences

## Abstract

Tomato (*Solanum lycopersicum* var. *cerasiforme*) is a nutritionally valuable crop, yet yellow cherry tomato cultivation faces yield limitations under protected environments. This study investigated the effects of gibberellic acid (GA_3_) and benzylaminopurine (BAP) on growth, yield, and nutrient components in greenhouse-grown yellow cherry tomatoes. Treatments included foliar applications of GA_3_ (25, 50, and 75 mgL^− 1^) and BAP (50 and 100 mgL^− 1^), sprayed on plant leaves at 14, 28, and 42 days after transplanting, before the flowering stage. GA_3_ at 75 mgL^− 1^ enhanced stem diameter (11.31 mm), branch number (22), and fruit biomass (1.13 g/fruit), raising yield by 93.8% over control, while BAP produced the highest yield increase (108.4%). Also, BAP increased chlorophyll content and leaf micronutrient retention (e.g., zinc: 0.859 mg kg^−1^). Fruit nitrogen and protein peaked under GA_3_ 25 mgL^− 1^ (3.15% N, 19.66% protein), whereas higher GA_3_ concentrations prioritized biomass over nutrient density. BAP reduced fruit shape index (1.49–1.64) and increased total soluble solids (TSS). Cytokinin-auxin interplay differentially regulates source-sink nutrient partitioning, with GA_3_ promoting vegetative nitrogen assimilation and BAP enhancing phloem-mediated micronutrient stabilization. These findings demonstrate that optimizing GA_3_ and BAP concentrations can balance yield and nutritional quality, providing actionable strategies for improving greenhouse cultivation of yellow cherry tomatoes.

## Introduction

Tomato (*Solanum lycopersicum* L., family *Solanaceae*) is one of the most important crops worldwide; it’s an important source of nourishment with a total production of approximately 162 million tons annually. Egypt has a production share of around 4.7% as one of the top 5 producers globally^1^. Tomatoes are grown under a wide range of climatic conditions. Recently, yellow tomato has become a fresh product in markets due to its high nutritional values when compared to red tomato, such as more calories, higher protein, fiber, and carbohydrates^2^. Among tomato varieties, the Cherry tomato (*Solanum lycopersicum var. cerasiforme*), which is a small-sized garden variety of tomato and is closely related to wild tomatoes^1^ has gained considerable consumer likeness due to its unique size, sweetness, and higher nutritional value^3,4^However, the yellow cherry tomato variety has a lower production rate than standard varieties and requires unique agronomical practices, such as controlled greenhouse production and application of phytohormones to enhance its yield and fruit quality.

Plant growth regulators (PGRs) are natural compounds that are excreted by plants or synthetically developed. These PGR substances have a wide range of engagements to accelerate and enhance physiological processes, including cell elongation and plant growth, functions like flowering and seed germination, and increase fruit size and quality^5,6^There are several PGRs, each with a specific molecular activity. Among the major ones are abscisic acid, auxins, gibberellins, and Cytokinins. The use of PGRs has resulted in some achievements in several fruit crops regarding fruit quality and growth^7–10^. For instance, Gibberellic acid (GA_3_) is one of the most well-known and widely used gibberellins, a class of diterpenoid plant hormones, which play a fundamental role in regulating growth and development. Its primary mechanism involves promoting both cell elongation and cell division, processes essential for tissue expansion and overall plant growth^11^. Different studies have documented the effects of exogenous GA_3_ application on various plant morphological traits^12^. A consistent and prominent effect is increased stem elongation, leading to taller plants. This is often accompanied by enhanced leaf expansion and size. Effects on branching can vary, with some reports indicating an increase, while impacts on root growth are also noted^13^. It is important to note that GA_3_ effects are often dose-dependent, with excessive concentrations potentially leading to undesirable outcomes like overly spindly growth or reduced root development^14^.

Benzylaminopurine (BAP), also known as 6-Benzyladenine (BA), is a synthetic cytokinin, a class of plant hormones primarily known for their role in promoting cell division (cytokinesis)^15^. The impact of BAP includes not only cell division but also nutrient assimilation, photosynthesis, stress responses, and the regulation of senescence^16,17^. BAP is also known to influence plant morphology. Its primary effect is the stimulation of cell division, promoting lateral bud growth, thereby overcoming apical dominance and therefore increasing the number of branches^18,19^.

Despite the established roles of gibberellin and cytokinin in regulating plant growth and fruit development^6,20^, and the increasing agricultural importance of cherry tomatoes (*Solanum lycopersicum var. cerasiforme*), a significant knowledge gap exists regarding the effects of exogenously applied BAP and GA_3_ on morphological characteristics and yield components of this variety, particularly yellow-fruited cultivars. Also, major limitations of greenhouse tomato production systems exist, such as water and nutrient management^21^, higher operational cost^22^, and varietal and technical suitability. Previous studies have often focused on standard red tomato cultivars^1,23–25^ and individual PGR effects, leaving the potential interplay between BAP and GA_3_ in yellow cherry tomatoes largely unexplored. Furthermore, optimal application strategies, including effective concentrations for maximizing desirable traits in this specific cultivar group, remain undetermined. In this regard, this study aimed to evaluate the effects of GA_3_ and BAP on the growth and yield characteristics of yellow cherry tomato plants cultivated under controlled greenhouse conditions to further optimize and reduce the cost in production systems.

## Results

### Effect of PGRs on different morphological and phenological traits

#### Growth traits

**Fig. 1:**
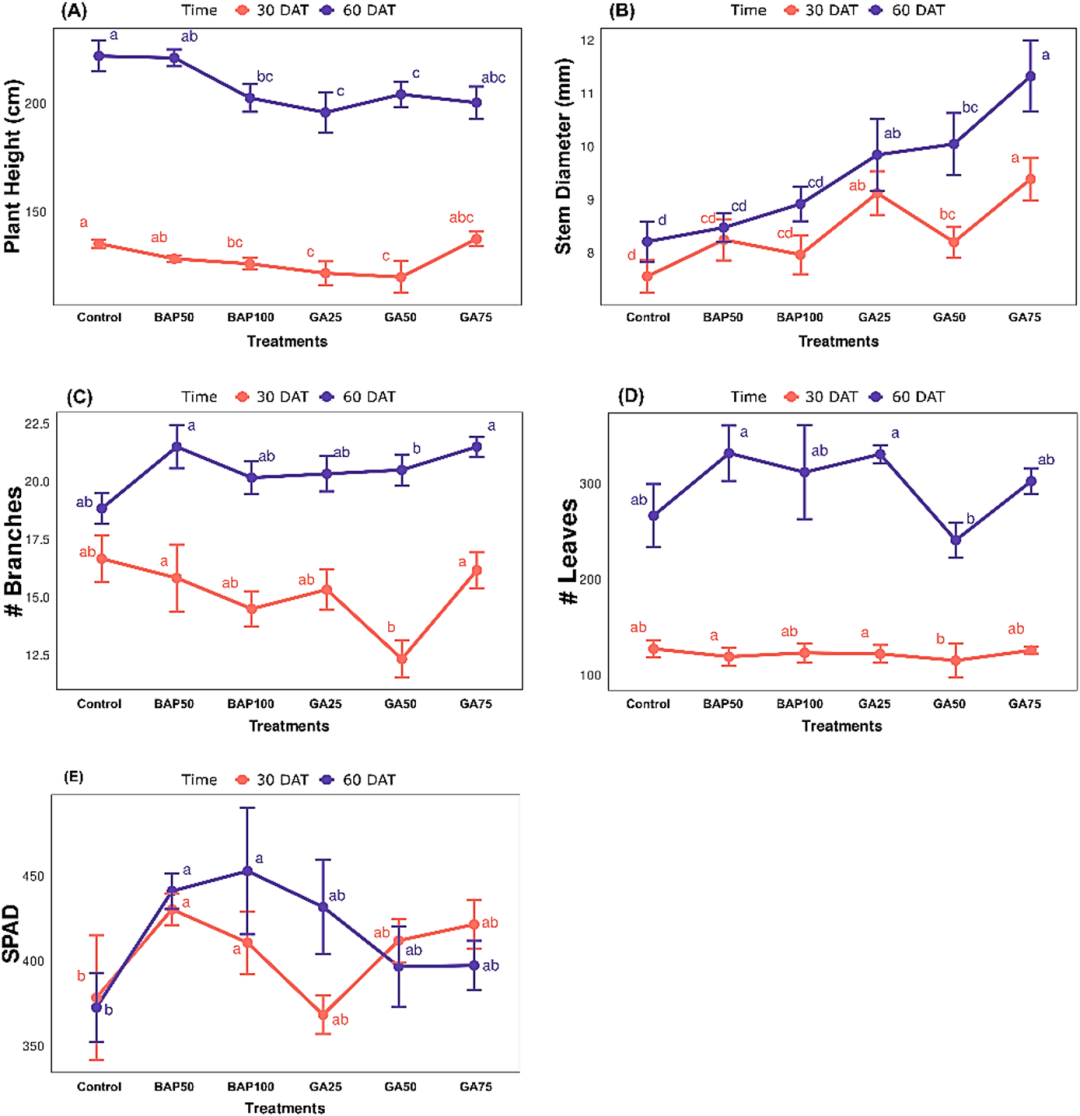
Effects of BAP and GA_3_ treatments on growth and physiological parameters of yellow cherry tomato plants at 30 and 60 days after transplanting (DAT). (**A**) Plant height (cm), (**B**) stem diameter (mm), (**C**) number of branches, (**D**) number of leaves, and (**E**) SPAD values were measured under different treatments: Control, BAP50, BAP100, GA25, GA50, and GA75. Data are presented as means ± standard error (*n* = 6). Different letters indicate statistically significant differences among treatments within each time point (*p* < *0.05*).

All treatments significantly (*p ≤ 0.05*) altered plant height and stem diameter. Stem diameter significantly increased across treatments for 30 and 60 days after transplanting (DAT); plant height was demonstrated to be statistically reduced across treatments; this reduction was increased in the application with GA75, as shown in Fig. 1A. Among the different treatments, GA75 showed the greatest average in stem diameter of 7.5 and 8.2 cm at 30 and 60 DAT, respectively. GA75 also slightly increased the plant height compared to the control after 30 DAT. Different treatments caused significant reductions in the number of branches. The lowest number of branches, with a mean of 0.9 branches, was recorded at the application of GA50 (Fig. 1C), while the number of leaves and SPAD values were not changed significantly across treatments (Table 1). Compared with the control at 60 DAT, the number of leaves increased in BAP 50, BAP 100, G25, and G75. Contrary to the aforementioned, GA50 recorded (240 leaves) lower than the control (266 leaves) (Fig. 1D). Among the different treatments, the control recorded the lowest SPAD values, 372.73, while the highest SPAD values at 30 and 60 DAT were recorded at the application of BAP50, 430.37, 441.12, and BAP100, 410.78, 452.92. On the other hand, SPAD values decreased in GA_3_ treatments at 60 DAT (Fig. 1E).

**Table 1:** Analysis of variance among agronomic traits assessed in yellow Cherry tomato under plant growth regulators treatment.

	SOV	d.f.	M.S.	V.*R*.	F pr.
Plant height	Treatment	5	702	3.920	0.003 **
	Time	1	18,655	104.234	1.43e−14 ***
	Treatment*time	5	336	1.878	**0.11219**
	Residual	58	179		
Stem diameter	Treatment	5	9.688	9.036	2.15e−06 ***
	Time	1	14.224	13.267	0.00578 ***
	Treatment*time	5	1.416	1.321	**0.268079**
	Residual	58	1.072		
Number of leaves	Treatment	5	4255	1.156	0.199
	Time	1	77,458	27.600	2.23e−06 ***
	Treatment*time	5	3868	1.378	0.246
	Residual	58	2806		
Number of branches	Treatment	5	9.49	2.069	0.0788
	Time	1	99.7	22.021	1.69e−05 ***
	Treatment*time	5	11.03	2.437	0.0451 *
	Residual	58	4.53		
SPAD	Treatment	5	5885	2.203	0.0661
	Time	1	5861	2.194	0.1439
	Treatment*time	5	3548	1.329	0.2650
	Residual	58	2671		

Statistical differences were evaluated using two-way ANOVA. SOV: source of variation, d.f: degrees of freedom, M.S.: mean square, V.R.: variance ratio, and F: F value.

SOV: source of variation, d.f: degrees of freedom, M.S.: mean square, V.R.: variance ratio, and F: F value.

**Table 2:** Analysis of variance among yield traits assessed in yellow Cherry tomato under plant growth regulators treatment.

Trait	SOV	d.f.	M.S.	V.*R*.	F pr.
Fruit weight	Treatment	5	32.458	9.290	0.000001***
	Residual	63	3.494		
Fruit number	Treatment	5	219.933	0.12622	0.985
	Residual	63	1742.411		
TSS	Treatment	5	0.456	0.911	0.506
	Residual	63	0.500		
Fruit shape index	Treatment	5	0.049	3.075	0.0151*
	Residual	63	0.016		
E. Diameter	Treatment	5	21.286	6.272	0.0001***
	Residual	63	3.394		
P. Diameter	Treatment	5	6.750	1.018	0.415
	Residual	63	6.630		

Statistical differences were evaluated using one-way ANOVA. E. Diameter: Equatorial diameter, P. Diameter: Polar Diameter.

SOV: source of variation, d.f: degrees of freedom, M.S.: mean square, V.R.: variance ratio, and F: F value.

#### Effects of PGRs on yield traits

**Fig. 2:**
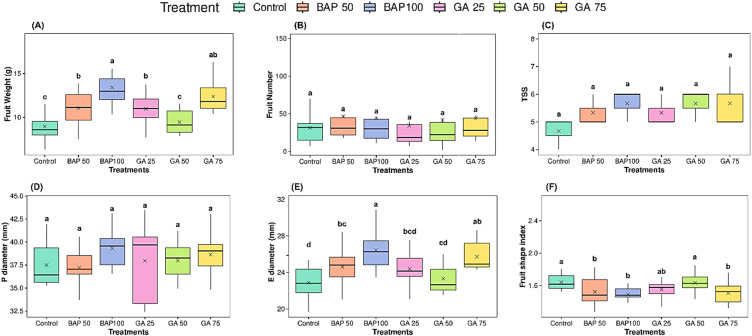
Effects of BAP and GA_3_ treatments on yellow cherry tomato fruit yield and quality parameters. (**A**) Fruit weight, (**B**) fruit number, (**C**) total soluble solids (TSS), (**D**) polar diameter, (**E**) equatorial diameter, and (**F**) fruit shape index of yellow cherry tomato fruits under different treatments: Control, BAP 50, BAP 100, GA 25, GA 50, and GA 75. Different letters indicate statistically significant differences among treatments for each parameter (*p ≤ 0.05*).

All treatments significantly (*p* ≤  *0.05)* changed fruit weight, which ranged from 8.95 g (control) to 13.4 g (BAP100) treatments. Among the different treatments, BAP100 showed the highest fruit weight across treatments. The application of BAP demonstrated an increase in fruit weight; this increase was significant in the continued treatment (Fig. 2A; Table 2). Similarly, the application of GA_3_ showed an increase in fruit weight at concentrations of GA25 and GA75 (11 g and 12.39 g, respectively), whereas lower values of 9.46 g were recorded at GA50 compared to GA25 and GA75 (Fig. 2A). There was a significant (*p ≤ 0.05*) variation in fruit shape index with values ranging from 1.49 to 1.64 across all treatments (Fig. 2F). The control treatment showed the highest mean value (1.64) compared to the BAP treatments and GA75. Conversely, the foliar application of GA_3_ and BAP had no significant effect on fruit number, total soluble solids (TSS), and polar diameter of fruits (Table 2; Fig. 2B and D). Fruit equatorial diameter significantly (*p ≤ 0.05*) increased due to the treatments, with the highest values observed in BAP100. The cumulative yield for plants was increasing with the application of GA_3_ and BAP (Table 3), where the highest yield across treatments was observed with the application of BAP 100 ~ (588 g/plant) and GA 75 ~ (547 g/plant), while the control treatment exhibited the lowest yield (282.2 g/plant).

**Table 3:** Cumulative fruit yield per plant across treatments.

Treatment	Cumulative yield (g/plant)	% Increase based on Control
Control	282.2	–
BAP 50	514.16	82.19%
BAP 100	588	108.36%
GA 25	375.6	33.09%
GA 50	395.8	40.25%
GA 75	547	93.83%

**Fig. 3:**
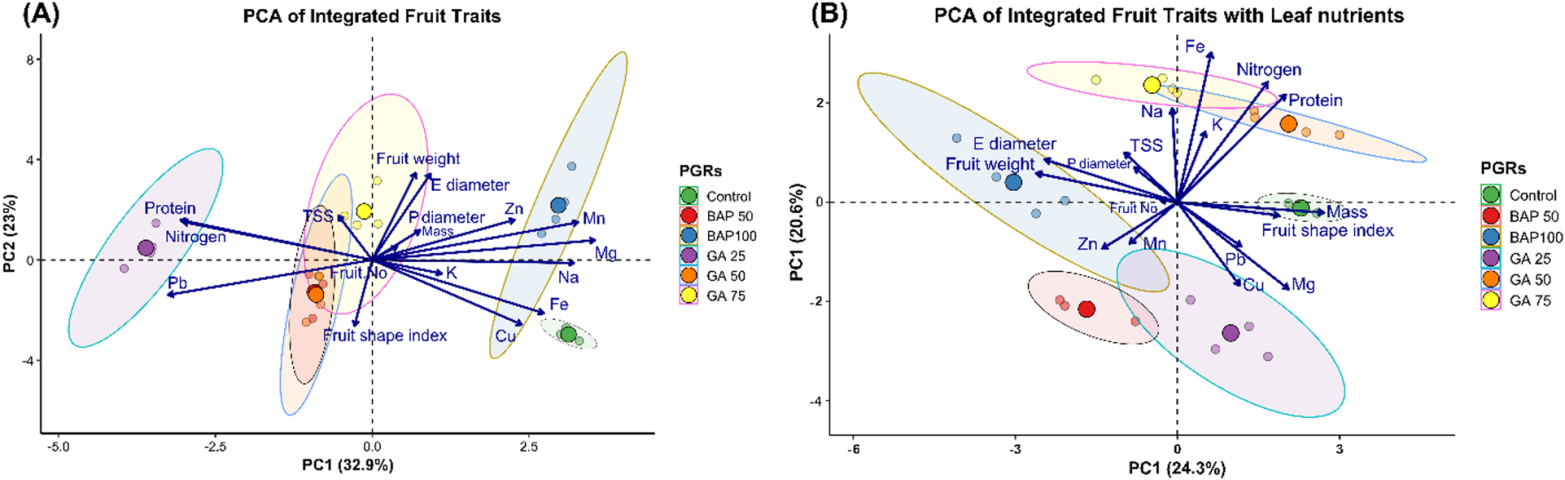
Principal component analysis (PCA) of integrated yellow cherry tomato fruit traits and leaf nutrient profiles across six plant-growth regulator (PGR) treatments. (**A**) PCA of fruit quality and morphology descriptors alone; (**B**) PCA integrating both fruit descriptors and leaf nutrient concentrations, Equatorial Diameter (E diameter) Polar Diameter (P diameter), Fruit Number (fruit No) Total Soluble Solids (TSS), Manganese (Mn), Sodium (Na), Potassium (K) Iron (Fe), Copper (Cu), Magnesium (Mg), Zinc (Zn), Lead (Pb). Ellipses denote 95% confidence intervals for each treatment group: Control (green), BAP50 (red), BAP100 (purple), GA25 (blue), GA50 (cyan), and GA75 (yellow).

In the PCA of fruit traits alone (Fig. 3A), the first two principal components explained 32.9% (PC1) and 23.0% (PC2) of the total variance among treatments. PC1 primarily separated treatments based on size-related traits, fruit weight, equatorial (E) and polar (P) diameters, and fruit number, positively, whereas protein content, total soluble solids (TSS), and leaf Pb content loaded negatively. Notably, GA75 clustered strongly on the positive PC1 axis, reflecting the highest average fruit weight and dimensions, while BAP100 and BAP50 fell on the negative side, indicating smaller fruits with higher TSS and protein. PC2 distinguished treatments by shape and micronutrient associations: control plants were characterized by more elongated fruit (higher shape index) and elevated leaf manganese (Mn) and zinc (Zn), whereas GA50 aligned with higher sodium (Na) and potassium (K) levels.

When leaf nutrient concentrations were incorporated (Fig. 3B), PC1 and PC2 accounted for 24.3% and 20.6% of variance, respectively, revealing a clearer nutrient–trait coupling. GA75 remained at the extreme positive end of PC1, now also associated with elevated leaf iron (Fe) and protein, underscoring its strong promotive effect on both biomass and nutritional quality. Conversely, BAP treatments (especially BAP100) clustered on the negative PC1 and PC2 quadrants, correlating with higher leaf lead (Pb) and copper (Cu) levels but reduced fruit size. Control and GA25 formed an intermediate group, positioned near zero on PC1 but separated along PC2 by differences in magnesium (Mg) and Mn content. Overall, the integrated PCA underscores that high-concentration GA treatments shift tomato plants toward larger, nutrient‐enriched fruits, while BAP treatments tend toward smaller fruits with altered micronutrient partitioning.

**Table 4:** Interactive effect of different concentrations of Benzylamino-purine (BAP) and gibberellic acid (GA_3_) on fruit and leaves nutrient components.

Tissue	Treatment	Mass (gm)	Nitrogen %	Protein %	Zn (mgL^− 1^)	Cu (mgL^− 1^)	Pb (mgL-1)	Mn (mgL^− 1^)	Fe (mgL^− 1^)	Mg (mgL^− 1^)	K (mgL^− 1^)	Na (mgL^− 1^)
Fruits	Control	1.06^b^	2.76^f^	17.14^f^	0.11^c^	0.02^a^	0.04^e^	0.2^b^	1.88^a^	8.71^a^	96.64^a^	28.63^b^
	BAP50	1^b^	2.88^c^	18.03^c^	0.04^f^	0.02^b^	0.05^c^	0.18^d^	0.64^c^	6.62^d^	87.64^f^	27.31^c^
	BAP100	1.01^b^	2.86^d^	17.85^d^	0.13^b^	0.02^c^	0.03^f^	0.29^a^	0.71^b^	8.73^a^	90.85^e^	29.09^a^
	GA25	1.01^b^	3.15^a^	19.66^a^	0.06^e^	0.01^e^	0.05^b^	0.13^f^	0.24^e^	6.22^e^	91.44^d^	25.35^e^
	GA50	1.05^b^	2.81^e^	17.58^e^	0.1^d^	0.01^d^	0.05^a^	0.15^e^	0.34^d^	7.23^c^	93.63^c^	25.07^f^
	GA75	1.13^a^	2.93^b^	18.31^b^	0.15^a^	0.01^e^	0.04^d^	0.18c	0.24^f^	8.33^b^	95.56^b^	25.43^d^
Leaves	Control	1.006^a^	3.94^b^	24.67^b^	0.329^f^	0.011^e^	0.057^c^	1.315^b^	0.639^c^	9.617^d^	92.172^e^	36.596^f^
	BAP50	1.001^d^	3.66^c^	22.89^c^	0.859^a^	0.013^c^	0.048^d^	2.071^a^	0.399^f^	9.629^c^	89.582^f^	38.421^c^
	BAP100	1.001^d^	3.55^d^	22.02^d^	0.549^c^	0.012^d^	0.039^f^	0.833^d^	0.621^d^	9.218^f^	93.062^d^	37.899^d^
	GA25	1.003^c^	3.54^d^	22.72^c^	0.413^d^	0.018^a^	0.062^a^	0.512^f^	0.423^e^	10.216^a^	95.155^b^	36.712^e^
	GA50	1.005^b^	4.07^a^	25.46^a^	0.566^b^	0.015^b^	0.041^e^	0.623^e^	0.712^a^	9.855^b^	93.546^c^	39.821^a^
	GA75	1.001^d^	4.01^ab^	25.10^a^	0.382^e^	0.012^d^	0.059^b^	0.92^c^	0.692^b^	9.412^e^	97.022^a^	38.452^b^

Each value is a mean of three replicates; values sharing different superscript letters indicate significant differences (*p ≤ 0.05*) among treatments. Manganese (Mn), Sodium (Na), Potassium (K), Iron (Fe), Copper (Cu), Magnesium (Mg), Zinc (Zn), Lead (Pb).

## Discussion

This study evaluated the effect of plant growth regulator application on growth and yield traits of yellow cherry tomato at 30 and 60 days after transplanting (DAT). Overall, certain traits exhibited significant variation under different concentrations of plant growth regulators (Table 1). At 30 DAT, plant height and number of leaves (Fig. 1A and C) showed lower performance to no effect with the treatments. Although GA3 should increase plant height, the observed reduction in height after GA3 application in this study could be due to the interacting effects of GA3 and abscisic acids^26^. This antagonistic balance is a crucial mechanism for integrating a plant’s internal growth signals with environmental conditions, where a higher ABA-to-GA3 ratio generally leads to decreased growth and height to conserve resources^27^. High levels of GA accelerate metabolic processes and sink demand in fruits^28,29^. We hypothesize that the trigger assimilates towards early flowering and fruiting rather than elongation; in addition, the number of branches decreased. Exogenous applications of GA_3_ and BAP have limited effects at early growth stages due to hormonal balances and are not optimal for strong growth responses^30^. Research consistently showed that the application of cytokinin at early growth stages with young growing shoots has no effect on promoting lateral buds^31^ Although 50 mg/L and 100 mg/L were chosen based on previous studies^32,33^Further studies are needed to elucidate much lower concentrations of BAP for this tomato variety, since varieties differ in their response to PGRs., while significantly at 60 DAT (Table 1) number of branches (≃ 22) and stem diameter (11.31 cm) exhibited higher performance with the application of GA_3_ at 75 mgL^− 1^ and BAP at 50 mgL^− 1^ (Fig. 1B and C)^31^, which resulted significantly in Table 1, aligning with^34^As the gibberellins promote plant growth by stimulating shoot elongation, seemingly these traits increased with the application of GA. Higher measurements of vegetative growth were found at the application of Gibberellins GA^35,36^. In essence, GA upregulates the genes involved in cell cycle progression, particularly G2-M phase, such as cyclin-dependent kinase 1 (CDK1)^37^, which regulates the transition between the G2 phase and mitosis^38^. Similarly, BAP increased the number of branches, leaves, and chlorophyll content (Fig. 1C–E). This result can be attributed to the fact that cytokinins, such as BAP, are recognized for their ability to reduce apical dominance and promote the growth of axillary buds^39^, which can interact with other hormones such as brassinosteroids and strigolactones to regulate the expression of genes like BRANCHED1, which is a key inhibitor of bud outgrowth^19^. Chlorophyll content consistently increased with the concentration of BAP and decreased with the application of GA, as mentioned by^40^, who demonstrated that the BAP application impacted the chlorophyll content while decreasing with GA; therefore, BAP led to higher chlorophyll accumulation in leaves, triggered by upregulation of chlorophyll biosynthetic genes^41^.

GA_3_ and BAP influenced fruit characteristics in cherry tomato (Fig. 2). Our findings demonstrated that the application of both GA_3_ and BAP significantly affected fruit weight and equatorial diameter, resulting in higher performance (Fig. 2A and E). This observation aligns with^42^, who reported that elevated cytokinin levels promote fruit thickening and enlargement. In this study, the fruit shape index ranged from 1.49 to 1.64. GA_3_ treatment produced a slight but significant increase in fruit shape index, which resulted in more elongated fruits (Fig. 2F). This outcome is consistent with research showing that exogenous gibberellin promotes fruit elongation^17,42^. Conversely, the application of BAP resulted in a decrease in the fruit shape index. This observed reduction in the fruit shape index corresponds to an increase in the equatorial diameter of fruit, which is likely influenced by the application of BAP^43^. The number of fruits increased slightly with BAP (Fig. 2B). Higher concentrations (600 ppm) of BAP increased fruit sets per plant, and GA_3_ increased fruit numbers per plant^44^. Previous studies have shown that GA_3_ induces parthenocarpy^45,46^. Gibberellins promote cell division in ovaries, leading to more fruit formation without pollination, with more locules^46^. TSS were found to be affected by the application of PGRs (Fig. 2C), with the highest value observed in the application of BAP100, GA50, and GA75. Similar observations were reported by^20^, who found that GA increased TSS in fruits.

PGR treatments across both leaves and fruits induced distinct alterations in biomass and nutrient profiles. In fruits, the highest biomass was recorded under GA75 (1.13 g), which significantly exceeded the control and BAP treatments. In contrast, treatments with GA25 and BAP did not lead to a remarkable increase (Table 4). The concentration of nitrogen (N) and protein in fruits peaked under GA25 at (3.15%) N and (19.66%) protein, consistent with findings that GA_3_ enhances nitrogen remobilization and nitrate reductase activity when combined with optimal nitrogen levels^25–48^. GA stimulates nitrogen metabolism through enhanced enzyme activity in cellular compartments, promoting amino acid synthesis and protein accumulation in developing tissues while simultaneously increasing nitrate transport capacity in both xylem and phloem^48–50^. Higher GA_3_ concentrations (75 mg L^− 1^) prioritized biomass over compositional quality, a pattern observed in previous studies where excessive GA_3_ led to nutrient dilution despite yield gains^48,51^. Fruit zinc (Zn) peaked under GA75 (0.15 mg kg^−1^) but minimized under BAP50 (0.04 mg kg^−1^), reflecting cytokinin role in restricting Zn translocation via vacuolar sequestration, as demonstrated in tomato regeneration studies^52,53^, by enhancing tonoplast transporter activity and cytosolic chelator production, which represents a dynamic cellular homeostasis mechanism where the vacuole functions as a buffering compartment, actively consuming cellular resources and maintaining ionic balance^54^ Manganese (Mn) and iron (Fe) levels increased under BAP100 and GA75, respectively, highlighting differential micronutrient partitioning influenced by PGRs^53,55^. Magnesium (Mg) and potassium (K) decreased under GA25, likely due to dilution effects in rapidly expanding tissues^48,51^. In leaves, BAP50 increased Zn (0.859 mg kg^−1^) and Mn (2.071 mg kg^−1^), corroborating BAP’s efficacy in enhancing micronutrient retention through phloem regulation^53,55^. GA25 and GA50 elevated leaf Fe (10.216–9.855 mg kg^−1^) and K (93.546 mg kg^−1^) levels, consistent with GA_3_’s stimulation of nutrient transporter activity^56,57^. Peak leaf N (4.07%) and protein (25.46%) under GA 50 underscore gibberellins’ role in promoting nitrogen assimilation, as seen in trials combining GA_3_ with nitrogen fertilization^48,57^. GA3 functions as a signal between various plant organs, connecting root physiology with the nutritional needs of the shoots, thereby supporting overall plant homeostasis^58,59^. For BAP, it influences nutrient homeostasis in plants by modulating nutrient uptake, translocation, and the regulation of metabolic pathways. It also regulates proteins involved in carbohydrate and energy metabolism, affecting protein synthesis and destination, and thus directly impacts plant growth and development by influencing internal nutrient distribution and utilization^60^. Collectively, the micro and macronutrient fluctuations observed reflect a homeostatic mechanism that plants employ to maintain optimal function under treatments. These physiological responses represent integrated cellular mechanisms where plants regulate nutrient distribution through complex interactions between hormone signaling, transport protein activity, cell wall mechanics, and source-sink relationships. Published work confirms that fine-tuning PGR dosage is critical for balancing tomato yield, fruit nutritional quality, and source–sink nutrient dynamics^61–63^.

## Materials and methods

### Plant material and experimental design

In the current study, Seeds of *Solanum lycopersicum* L. cultivar ‘Yellow Pear shaped’ were obtained from Horti Tops (Tuinplus, The Netherlands; catalogue no. 12830, seed lot #B062). Seeds were surface-sterilized in 1% sodium hypochlorite for 5 min, rinsed three times with sterile distilled water, and germinated in a pot experiment conducted in a standardized greenhouse at the Center of Applied Research and Environmental Sustainability (CARES), The American University in Cairo, from late August 2024 to mid-December 2025 i.e. from sowing, transplanting until final harvesting. Using a randomized complete block design of three blocks, each treatment consisted of 10 plants, where only one plant per pot was grown in a mixture of coco peat and perlite (volume ratio 3:1). Treatments included two plant growth regulators at various concentrations.

### GA_3_ and BAP treatments

Foliar applications of gibberellic acid (GA_3_) at 25, 50, and 75 mg L^− 1^, and benzylaminopurine (BAP) at 50 and 100 mg L^− 1^^64,65^, Distilled water was used for the control treatment, which was administered in the evening three times to the leaves of tomato plants at 14, 28, and 42 days after transplanting (DAT), before the flowering stage.

### Growth and yield parameters determination

Growth parameters were measured at 30 and 60 DAT. Yield and fruit quality data were collected at each harvest until the end of the experimental period. Plant heights were measured from the crown to the growing tip of the plant, stem diameter was measured using vernier calipers, the number of branches and leaves were counted and averages determined, SPAD was measured in the early morning using a chlorophyll meter (Apogee Instruments MC-100. A) total of 6 harvests was made, and the number of fruits per harvest per treatment was recorded, and averages were determined. Cumulative yield was calculated with this formula:

$${\text{Cumulative Yield }}\left( {{\text{g}}/{\text{plant}}} \right)={\text{Average number of Fruits per Plant}} \times {\text{Average Fruit Weight }}\left( {\text{g}} \right)$$

### Nutrient analysis

Three Samples of yellow cherry tomato fruits and leaves were thoroughly washed with deionized water to remove surface contaminants. Fruit and leaves nutrient contents were determined using microwave-assisted acid digestion and appropriate instrumental analysis. Briefly, samples (300 mg) were accurately weighed into DAK-60 K digestion vessels. Concentrated nitric acid (8.0 mL, 65% HNO_3_) was added to each vessel, and the mixture was carefully stirred with a Teflon rod. After a pre-reaction period of 10 min, the vessels were sealed and placed in a Berghof Speedwave Entry microwave digestion system (Berghof Products + Instruments GmbH, Germany). The digestion program consisted of four steps: (1) 170 °C for 5 min (10 min total, 90% power); (2) 200 °C for 3 min (15 min total); (3) 75 °C for 1 min (10 min total); and (4) cooling to 50 °C. After cooling to room temperature (~ 20 min), the vessels were carefully opened to avoid gas release. The resulting clear solutions were quantitatively transferred to volumetric flasks, and elements, such as zinc, iron, copper, manganese, sodium, magnesium, and potassium, were quantified using an Atomic Absorption Spectrophotometer, Pye Unican SP1900.

### Protein and nitrogen analysis

Total protein content was determined using the Kjeldahl method according to AOAC 981.10^66^. Samples with mass weights of 1–2 g were weighed into 300 mL digestion tubes. Two catalyst tablets (containing 4.98 g K_2_SO_4_ and 0.02 g CuSO_4_·5 H_2_O) and 15 mL of concentrated sulfuric acid (98%) were added to each tube. Blank samples were prepared similarly without sample material. Digestion was performed using a BUCHI Speed Digester K-439 (Büchi Labortechnik AG, Switzerland) connected to a BUCHI Scrubber K-415 for acid fume neutralization. The temperature program consisted of preheating to 350 °C; step 1 at 350 °C for 10 min; step 2 at 550 °C for 15 min; step 3 at 490 °C for 65 min; followed by cooling for 30 min. After cooling, the digested samples were processed using a BUCHI KjelMaster K-375 (Büchi Labortechnik AG, Switzerland). Distillation was performed with the addition of 50 mL of water and 80 mL of sodium hydroxide solution (32%), with a reaction time of 5 s. The distillation was conducted in fixed-time mode for 300 s at 100% steam output with a stirrer speed of 5. The ammonia released was collected in 50 mL of 4% boric acid solution. Titration was performed using 0.05 M sulfuric acid or hydrochloric acid with potentiometric or colorimetric endpoint detection at pH endpoint measurement mode. The protein content was calculated according to the following formula:

$${\text{Protein }}\% =\left( {{\text{VA}} - {\text{VB}}} \right) \times {\text{1}}.{\text{4}}00{\text{7}} \times {\text{N}} \times {\text{6}}.{\text{25}}/{\text{sample weight}}$$

where VA and VB = volumes of standard acid required for sample and blank titration, respectively; 1.4007 is the milliequivalent weight of nitrogen; *N* × 100(%) = normality of the standardized acid; and 6.25 is the protein conversion factor for plant materials.

All analyses were performed in triplicate to ensure the reliability and reproducibility of results.

### Statistical analysis

Analysis of variance (ANOVA) and visualizations were conducted using R (v4.4.3, R Core Team 2025) in RStudio (v2024.12.1). Mean differences were compared by an LSD test using the agricolae R package (v1.3-7)^69^. Differences of *p* ≤ 0.05 were considered. For visualizing the descriptive statistics of the traits, we used box and whisker plots and line charts. These visualizations were created using the ggplot2 package^70^.

## Conclusion

In conclusion, this study shows that plant growth regulators (GA_3_ and BAP) can improve the growth, yield, and quality of yellow cherry tomatoes; for instance, using 100 mg/L BAP or 75 mg/L GA_3_ improved fruit weight, while exhibiting a lower number of branches and shorter plant height, which may reduce the labor production cost. However, a lower dose, 25 mg/L GA_3,_ led to higher fruit nitrogen and protein. Also, BAP treatments improved chlorophyll and helped leaves keep their nutrients, but reduced zinc transfer to the fruit.

We suggest that, to get the best results, growers should choose treatments based on what they want to improve. For the highest yield and branch growth, 75 mg/L GA_3_ is best. For better fruit nutrition without lowering yield, 25 mg/L GA_3_ is recommended. Applying 50–100 mg/L BAP helps plant health and leaf nutrients but requires monitoring fruit zinc levels.

These results are from greenhouse tests with one yellow cherry tomato variety, so they may not apply to all production systems. Future studies should look at how GA_3_ and BAP interact at the molecular level and confirm these findings in field conditions to improve their practical use.

Future research should investigate the molecular mechanisms underlying GA_3_-BAP crosstalk, particularly their influence on auxin signaling and nutrient transporter expression, to enhance strategies for sustainable tomato production. Field trials under complex environmental conditions are recommended to validate these findings and optimize protocols for commercial scalability.

## Data Availability

Datasets generated during this study are available from the corresponding author upon reasonable request.
